# Respiratory microorganisms in acute pharyngitis patients: Identification, antibiotic prescription patterns and appropriateness, and antibiotic resistance in private primary care, central Malaysia

**DOI:** 10.1371/journal.pone.0277802

**Published:** 2022-11-17

**Authors:** Zhuang Mian Bo, Wei Keat Tan, Christina Shook Cheng Chong, Munn Sann Lye, Seshatharran Parmasivam, Shu Ting Pang, Shobha Elizabeth Satkunananthan, Hui Yee Chong, Ameen Malek, Batool Abdulhafidh Ahmed Mohammed Al-khazzan, Benedict Lim Heng Sim, Christopher Kwok Chong Lee, Renee Lay Hong Lim, Crystale Siew Ying Lim

**Affiliations:** 1 Faculty of Applied Sciences, Department of Biotechnology, UCSI University, Cheras, Kuala Lumpur, Malaysia; 2 Faculty of Medicine and Health Sciences, Formerly Department of Community Medicine, Universiti Putra Malaysia; 3 Department of Medicine, Hospital Sungai Buloh, Selangor, Malaysia; Defense Threat Reduction Agency, UNITED STATES

## Abstract

Acute pharyngitis (AP) is a common reason for private primary care consultations, thus providing an avenue for widespread antibiotic intake among the community. However, there is limited data on the antibiotic prescription appropriateness and resistance information in the Malaysian private primary care setting, therefore, this study aimed to investigate the prevalence of isolated viruses and bacteria, antibiotic resistance patterns, antibiotic prescription patterns and appropriateness by general practitioners (GPs) and factors affecting antibiotic resistance and antibiotic prescription patterns. To investigate, a cross-sectional study was conducted among 205 patients presenting with AP symptoms at private primary care clinics in central Malaysia from 3^rd^ January 2016 to 30^th^ November 2016. Throat swabs were collected from 205 AP patients for two purposes: (i) the detection of four common respiratory viruses associated with AP via reverse-transcription real-time PCR (qRT-PCR); and (ii) bacterial identification using matrix-assisted laser desorption/ionisation time-of-flight mass spectrometry (MALDI-TOF MS). Bacterial isolates were then subjected to antibiotic susceptibility screening and McIsaac scoring was calculated post-prescription based on GP selection of criteria. Generalized estimating equations analysis with multiple logistic regression was conducted to identify factors associated with presence of virus and antibiotic prescription. The results showed that 95.1% (195/205) of patients had at least one of the four viruses, with rhinovirus (88.5%) being the most prevalent, followed by adenovirus (74.9%), influenza A virus (4.6%) and enterovirus (2.1%). A total of 862 non-repetitive colonies were isolated from the culture of throat swabs from 205 patients who were positive for bacteria. From a total of 22 genera, *Streptococcus* constitutes the most prevalent bacteria genus (40.9%), followed by *Neisseria* (20%), *Rothia* (13.0%), *Staphylococcus* (11%) and *Klebsiella* (4.9%). Only 5 patients carried group A beta-hemolytic streptococci (GABHS). We also report the presence of vancomycin-resistant *S*. *aureus* or VRSA (n = 9, 10.1%) among which one isolate is a multidrug-resistant methicillin-resistant *S*. *aureus* (MDR-MRSA), while 54.1% (n = 111) were found to carry at least one antibiotic-resistant bacteria species. Application of the McIsaac scoring system indicated that 87.8% (n = 180) of patients should not be prescribed antibiotics as the majority of AP patients in this study had viral pharyngitis. The antibiotic prescription appropriateness by applying post-prescription McIsaac scoring was able to rule out GABHS pharyngitis in this sample with a GABHS culture-positive sensitivity of 40% (n = 2/5) and specificity of 90% (180/200). In conclusion, antibiotic-resistant throat isolates and over-prescription of antibiotics were observed and McIsaac scoring system is effective in guiding GPs to determine occurrences of viral pharyngitis to reduce unnecessary antibiotic prescription.

## Introduction

Antibiotic resistance has predominantly been a clinical problem in hospital settings, but data from over the past two decades showed that antibiotic resistance in bacteria is spreading and rising in the community, and subsequently increase of community-acquired infections that are more difficult to identify and treat [1–3]. Moreover, it is important to note that 80% of antibiotic consumption takes place in the community with 20% to 50% being inappropriately used [4].

Upper respiratory tract infections (URTIs) account for 60% of all antibiotic prescriptions in the primary care setting [5] and is thus one of the most common reasons for visits to primary care physicians [6–8]. Unfortunately, high rates of antibiotic prescription have been reported for treatment of URTIs around the world [9,10]. This pattern of URTI diagnosis and antibiotic prescription in the primary care setting is also seen in Malaysia [11,12]. In Malaysia, public primary healthcare is mainly subsidized by the government while the private healthcare services are fee for services [13]. It was reported that the most common antibiotic groups prescribed in Malaysian primary care clinics are penicillins (amoxicillin, ampicillin, ampicillin-sulbactam, co-amoxiclav, bacampicillin, cloxacillin, penicillin V), cephalosporins (cefaclor, cephadroxil, cephalexin, cephalosporin (unspecified), macrolides (azithromycin, clarithromycin, erythromycin, roxithromycin, quinolones (ciprofloxacin, norfloxacin, ofloxacin) and tetracyclines (doxycycline, tetracycline) [14]. Acute pharyngitis (AP), or sore throat, is a community-based affliction with one of the highest rates of inappropriate antibiotic prescription in Malaysia [15]. Also, studies have shown that inappropriate prescription of antibiotics is more often seen in private practitioners than public practitioners [16]. Thus, clinical misuse and incorrect prescriptions of antibiotics may result in the resistance in bacterial infection that pose potential clinical complications to patients. Although globally studies have shown that only a minority of AP aetiology is attributable to group A beta-hemolytic streptococci (GABHS) or commonly known as *Streptococcus pyogenes* [17–19], the prevalence of GABHS as the aetiological agent of acute pharyngitis and the adequacy prescriptions of antibiotics for AP patients in the Malaysian private primary care setting are not known.

The human upper respiratory tract is a primary ecological site for various bacteria including members of the phyla Firmicutes, Actinobacteria, Bacteroidetes, Proteobacteria and Fusobacteria [20]. Colonization of bacterial pathogens is the first step before causing an infection, therefore, the constant exposure to antibiotics may influence bacterial colonization and favors the selection of resistant bacteria [21]. High prevalence of resistant *S*. *aureus* isolates was detected in nasal and throat of healthy food handlers and community-acquired methicillin-resistant *S*. *aureus* (CA-MRSA) was found to be able to remain persistently in human throat [22,23]. This points to a growing suspicion that antibiotic resistance spread in next decade will be primarily from human-to-human within the community via the oral route. With AP being one of the most common reasons for private primary care consultations, this provides an avenue for widespread antibiotic intake among the community.

Considering that GABHS pharyngitis is the most common reason for the indication of antibiotics, scoring systems such as the Centor criteria and McIsaac score have been developed to predict the likelihood of GABHS pharyngitis with respect to aid in antibiotic prescribing [24]. The risk classification for GABHS pharyngitis using the McIsaac scoring system can then be correlated to the recommendation of treatment for patients with each score [25]. According to the Clinical Practice Guidelines for Sore Throat Management, the possibility of patients having GABHS infection increases as the total score increases based on five criteria: age, tonsillar swelling or exudates, anterior cervical lymphadenopathy, absence of cough, and body temperature >38°C. Antibiotic therapy may be initiated in patients with a score of 3 points and above in Malaysia [11].

There is a dearth of information on antibiotic resistance among the general community in Malaysia especially patients who receive medical care from private primary healthcare providers for infectious diseases. Therefore, this study aims to investigate the prevalence of isolated viruses and bacteria, resistance patterns of pathogens found in human throat, antibiotic prescription patterns and appropriateness by GPs, factors affecting antibiotic resistance and antibiotic prescription patterns in AP patients.

## Methods

### Study design and settings

This cross-sectional study was conducted at a total of 17 private primary care clinics in the Klang Valley which consists of the urban areas of the state of Selangor as well as the federal territories of Putrajaya and Kuala Lumpur (also the capital of Malaysia) using convenience sampling. The minimum sample size was 187, calculated using Daniel’s formula [26], N = Z^2^ (P x q)/ d^2^. Considering: estimated prevalence of bacterial acute pharyngitis in adults [27] of 14.2% (P), with a 95% confidence interval (1.96) and a margin of error at 5%, q = 1–0.142 = 0.858.

### Ethical approval and sampling

Approval to conduct this study was granted by the Medical Research and Ethics Committee (MREC), Ministry of Health, Malaysia (reference number KKM/NIHSEC/P15–847), with the National Medical Research Register (NMRR) Approval No. NMRR-15-928-25596. Written informed consent was obtained from all participants. Convenience sampling was conducted from 3^rd^ January 2016 to 30^th^ November 2016. Convenience sampling was used in this study as this method is least expensive and able to produce results quickly. In addition, primary care clinics are the convenient sites for recruitment given the population of interest with sore throat always warrant a visit to the primary care clinic. Furthermore, private clinics provide more than 71.9% (7336 out of 10,198 health clinics) of primary care services for general population in Malaysia [28].

General practitioners (GPs) were each given an information sheet about this research study and materials for participants comprising of (i) patient information sheet and patient consent form; (ii) patient questionnaire, and (iii) two throat swabs, one for bacterial identification (Becton Dickinson Diagnostics, USA) and the other for virus detection (Greiner Bio-One), together with their respective transport media.

### Patient recruitment criteria

Eligible patients were Malaysians aged 18 years old and above with signs and symptoms of sore throat with fever. Patients who had antibiotic treatment within the past 2 weeks, had been hospitalized within the last 30 days, who had complicated pharyngitis such as peritonsillar abscesses (PTA), Lemierre disease or Vincent’s angina, immunosuppression or history of acute rheumatic fever and severe comorbidity were excluded from the study. GPs were requested to complete the patient questionnaire including patient’s demographics, history and duration of sore throat with fever within last 12 months, current throat infection, history and duration of antibiotics use within last 12 months, whether patients had finished the course of antibiotics prescribed in last sore throat infection, any antibiotics prescribed for current throat infection, type and dosage of antibiotics given and patients’ clinical symptoms and signs of current sore throat infection. The questionnaire also contained the 5 McIsaac criteria, however the antibiotic prescriptions by GPs was done based on sign and symptoms and the McIsaac scoring was calculated post-prescription based on GP selection of criteria. Throat swabs were taken by GPs by swabbing both sterile cotton swabs from the right tonsillar area across the posterior pharynx to the left tonsillar area and back across to the right tonsillar area in each patient. One of the swabs (for bacterial identification) was immediately stored in Amies transport medium (Becton Dickinson Diagnostics, USA) at room temperature whereas the other swab (for viral detection) was stored in phosphate buffered saline at 4°C, both were transported and processed at the laboratory within 24 hours of collection.

### Viral nucleic acid extraction and identification via real-time PCR

Viral DNA and RNA were co-extracted from the viral swabs using the GeneJET Viral DNA and RNA Purification Kit (Thermo Fisher Scientific, USA). Reverse transcription was carried out immediately using the SensiFAST cDNA synthesis kit (Bioline, Germany) according to the manufacturer’s instructions. For each sample, reverse transcription was performed in a 40 μL reaction volume containing 2 μL of reverse transcriptase, 8 μL of 5x TransAmp Buffer and 30 μL of extracted nucleic acids. Real-time PCR was performed in a StepOnePlus^TM^ Real-Time PCR System (Thermo Fisher Scientific, USA). A total of 4 human respiratory viruses were detected, including human adenovirus (hAdv), human rhinovirus (hRV), influenza A virus (FluA) and human enterovirus (hEV). For every 20 μL reaction (one reaction for each virus), consist of 10 μL of 2x SensiFAST Probe Hi-ROX Mix (Bioline, UK), 0.8 μL of each 10 μM virus forward and reverse primer, 0.2 μL of 10 μM FAM-labeled virus probe, 0.8 μL of each 10 μM *GAPDH* (human glyceraldehyde 3-phosphate dehydrogenase as the internal control) forward and reverse primer, 0.2 μL of 10 μM JOE-labeled *GAPDH* probe (all as in Table 1), 2 μL of reverse transcription reaction and 4.4 μL of UltraPure^TM^ DNase/RNase-Free water (Thermo Fisher Scientific, USA). No-template controls were included by substituting the reverse transcription reaction with UltraPure™ DNase/RNase-Free water. AmpliRun^®^ Enterovirus 71 RNA, Amplirun® Novel Influenza A H1N1 RNA control, Amplirun® Rhinovirus RNA control, Amplirun® Adenovirus DNA control were used as the positive controls. A FAM-positive signal (Ct value) indicated a positive sample (presence of a virus), while a sample was considered negative (absence of a virus) with a negative FAM signal (no Ct value) but positive JOE signal. All independent PCR reactions were performed in technical triplicates to obtain average cycle threshold (Ct) values using a StepOnePlus^TM^ Real-Time PCR System (Thermo Fisher Scientific, USA).

**Table 1:** Details of primers and probes for real-time PCR of four respiratory viruses and internal control.

Virus	Primer and probe sequence^#^	Product size (bp)	Target gene	Reference	qPCR Cycling conditions
Human adenovirus (hAdv)	5’-GGACGCCTCGGAGTACCTGAG-3’ 5’-ACIGTGGGGTTTCTGAACTTGTT-3’ **5’-FAM-CTGGTGCAGTTCGCCCGTGCCA-BFQ-3’**	96	Hexon region	[29]	95°C (3min), 45 cycles of 95°C (10s), 55°C (30s) and 72°C (15s)
Human rhinovirus (hRV)	5’-TCCTCCGGCCCCTGAAT-3’ 5’-GAAACACGGACACCCAAAGTAGT-3’ **5’-FAM-YGGCTAACCYWAACCC-BFQ-3’**	120	Polyprotein gene	[30]	95°C (2min), 45 cycles of 95°C (10s) and 65°C (25s)
Influenza A virus (FluA)	5’-GACCAATCCTGTCACCTCTGAC-3’ 5’-AGGGCATTCTGGACAAATCGTCTA-3’ **5’-FAM-TGCAGTCCTCGCTCACTGGGCACG-BFQ-3’**	106	Matrix protein	[31]	95°C (2min), 45 cycles of 95°C (10s) and 65°C (25s)
Human enterovirus (hEV)	5′-TCCTCCGGCCCCTGA-3’ 5′-RATTGTCACCATAAGCAGCCA-3’ **5′-FAM-CGGAACCGACTACTTTGGGTGWCCGT-BFQ-3’**	156	Polyprotein gene	[32]	95°C (2min), 45 cycles of 95°C (10s) and 65°C (25s)
**Internal control**					
Human glyceraldehyde 3-phosphate dehydrogenase (GAPDH)	5’-GAAGGTGAAGGTCGGAGT-3’ 5’-GAAGATGGTGATGGGATTTC-3’ **5’-JOE-CAAGCTTCCCGTTCTCAGCC-BFQ-3’**	226	*GAPDH*	[33]	As for the respective viruses

^#^Probe sequences are indicated in bold.

### Bacterial isolation and identification

Throat swabs for bacterial identification were inoculated on Tryptic Soy Agar with 5% sheep’s blood (TSAB) (Isolab Sdn Bhd, Malaysia) and incubated for 24 hrs at 37°C with 5% CO_2_. One to five single colonies with distinct morphologies (shape, colour, size and haemolytic pattern) were sub-cultured on TSAB agar and screened on the Microflex matrix-assisted laser desorption/ionisation-time of flight mass spectrometry (MALDI-TOF MS) Biotyper system (Bruker Daltonik GmbH, Germany) using the Bruker FlexControl software version 3.3 (Build 108) and analysed using the Bruker MALDI Biotyper Real Time Classification software (Version 3.1, Build 65).

### Antibiotic susceptibility testing (AST)

The bacterial isolates were subjected to AST using Kirby-Bauer disc diffusion method on Mueller Hinton agar with or without sheep blood and broth microdilution method using cation-adjusted Mueller Hinton broth (CAMHB) with or without lysed horse blood at 37°C with or without 5% CO_2_ according to specifications in the Clinical and Laboratory Standards Institute (CLSI) guidelines [34]. The ATCC strains *Staphylococcus aureus* (ATCC 25923 and ATCC 29213), *Escherichia coli* (ATCC 25922), *Streptococcus pneumoniae* (ATCC 49619) and *Pseudomonas aeruginosa* (ATCC 27853) were purchased and used as the reference strains in this study.

Briefly, three to five single colonies of each isolate were selected from LB or Nutrient agar streak plates (Merck, Germany) after overnight incubation at 37°C and transferred to 2 ml of 0.9% saline solution. The turbidity of bacterial suspension was adjusted to be equivalent to 0.5 McFarland to yield approximately 1.5 x 108 CFU/mL of *E*. *coli* ATCC 25922 for inoculum density standardization.

Antibiotic susceptibility testing was performed using selected antibiotics (See S1 Table). The result of Kirby-Bauer disc diffusion was obtained by measuring the inhibition zone diameter in millimetre (mm) and the result of broth microdilution test was obtained by determining the minimum inhibitory concentration (MIC) of antibiotic that inhibit the visible growth of bacteria. All AST were carried out in duplicates and results were interpreted as susceptible, intermediate and resistant category according to interpretive categories and zone diameter breakpoints (nearest whole mm) or MIC breakpoints (μg/mL) listed in CLSI guidelines [34].

### Data analysis

Descriptive statistics were used to describe patient’s demographic data (mean ± SD, percentages), antibiotic resistance patterns of bacterial clinical isolates and GPs’ antibiotic prescription patterns (reported as counts and percentages). Antibiotic prescription appropriateness was determined according to antibiotics prescribed by GPs based on GABHS-positive culture isolated from patients’ throat and McIsaac score (See S2 Table). Generalized estimating equations (GEE) for logistic regression (GEE Logit) was used to allow for dependence of outcome measures within clusters of patients and clinics. The outcome measures are respiratory viruses detected in patients nested within clinics, antibiotic resistance in bacteria isolated from patients nested within clinics and prescription practices of doctors nested within clinics. The unstructured working correlation matrix was specified. GEE Logit provided consistent estimates of B coefficients and standard errors to obtain odds ratios (OR) with 95% confidence intervals (95% CI) to identify factors associated with virus isolation, antibiotic resistance and antibiotic prescription. The alpha level of significance was set at 0.05. All statistical analyses were performed using SPSS v20.0 (IBM, Chicago, IL, USA).

## Results

### Characteristics of AP patients

Of the 219 pairs of throat swab collected from patients, 11 pairs of throat swabs were excluded from analysis due to leakage of transport medium from throat swabs stored in 1x PBS, while another 3 patients’ throat swabs were excluded because they were not Malaysian. Thus, a total of 205 throat swabs were processed for viral nucleic acid isolation and bacterial culture and identification respectively.

The mean age of 205 AP patients was 34.45±9.89 years and majority were middle-aged to elderly adults (≥31 years old). More than half of the subjects in this study (55.6%) were male and Chinese (69.3%) (Table 2). More than half of the AP patients were recorded to present with cough, malaise, headache and runny nose during primary care consultation (Table 3).

**Table 2:** Demography characteristics of AP patients (n = 205).

Patients’ Characteristics	Mean ± SD	Frequency, n (%)
**Age group (Years)**		
Overall	34.45± 9.89	
18–30		82 (40.0)
≥31		123 (60.0)
**Gender**		
Female		91 (44.4)
Male		114 (55.6)
**Ethnicity**		
Malay		36 (17.6)
Chinese		140 (68.3)
Indian		18 (8.8)
Others		11 (5.4)

**Table 3:** Sore throat symptoms and antibiotic prescription by GPs.

	Frequency, n (%)
**Presence of symptoms**	
Fever	76 (37.1)
Cough	124 (60.5)
Swollen	22 (10.7)
Exudate	65 (31.7)
Ulceration	13 (6.3)
Malaise	122 (59.5)
Runny nose	110 (53.7)
Hoarseness	51 (24.9)
Diarrhea	9 (4.4)
Gastrointestinal symptoms	10 (4.9)
Headache	101 (49.3)
**Antibiotic prescription by GPs**	120 (58.5)
**Types of antibiotics prescribed**	
**Penicillins** Amoxicillin Ampicillin	23 (19.2) 1 (0.8)
**Beta-lactam combination agents** Amoxicillin-clavulanate	52 (43.3)
**Macrolides** Azithromycin Erythromycin Clarithromycin Roxithromycin	6 (5.0) 9 (7.5) 7 (5.8) 3 (2.5)
**Cephalosporins** Cefuroxime Cefixime Cephalexin	3 (2.5) 1 (0.8) 9 (7.5)
**Fluroquinolones** Ciprofloxacin Levofloxacin	4 (3.3) 1 (0.8)
**Co-trimoxazole** Trimethoprim/sulfamethoxazole	1 (0.8)

### Respiratory viruses in AP patients

The majority of the AP in this study appear to be of viral aetiology. Among the 4 respiratory viruses investigated in this study of 205 AP patients, 95.1% (n = 195) had at least one respiratory virus. Human rhinovirus (hRV, n = 172, 88.5%) was the most frequently detected virus followed by human adenovirus, (hAdV, n = 146, 74.9%), influenza A (FluA, 4.6%, n = 9) and human enterovirus (hEV, 2.1%, n = 4). Out of the 195 patients in which at least one viruses was detected, the majority (70.3%, n = 137/195) had rhinovirus and adenovirus co-infections, while the presence of either adenovirus or rhinovirus was detected in 4.6% (n = 9) and 17.9% (n = 35) of patients. Patients’ age, gender and ethnicity were not significantly associated with presence of viruses detected in AP patients (Table 4).

**Table 4:** Risk factors associated with respiratory virus using Generalized Estimating Equation Logit modeling.

		B coefficient	Std. Error	Sig.	Exp (B)	95% Confidence Interval	
						Lower	Upper
**Age**		-0.001	0.351	0.984	1.0	0.933	1.070
**Gender**	**Female**	0.528	0.549	0.337	1.7	0.578	4.971
	**Male**				1		
**Ethnic**	**Malay**	0.365	0.393	0.353	1.4	0.667	3.109
	**Chinese**	1.270	0.812	0.118	3.6	0.725	17.467
	**Others**				1		

*Note*. *p<0.05.

### The pharyngeal microbiome of AP

**Fig 1:**
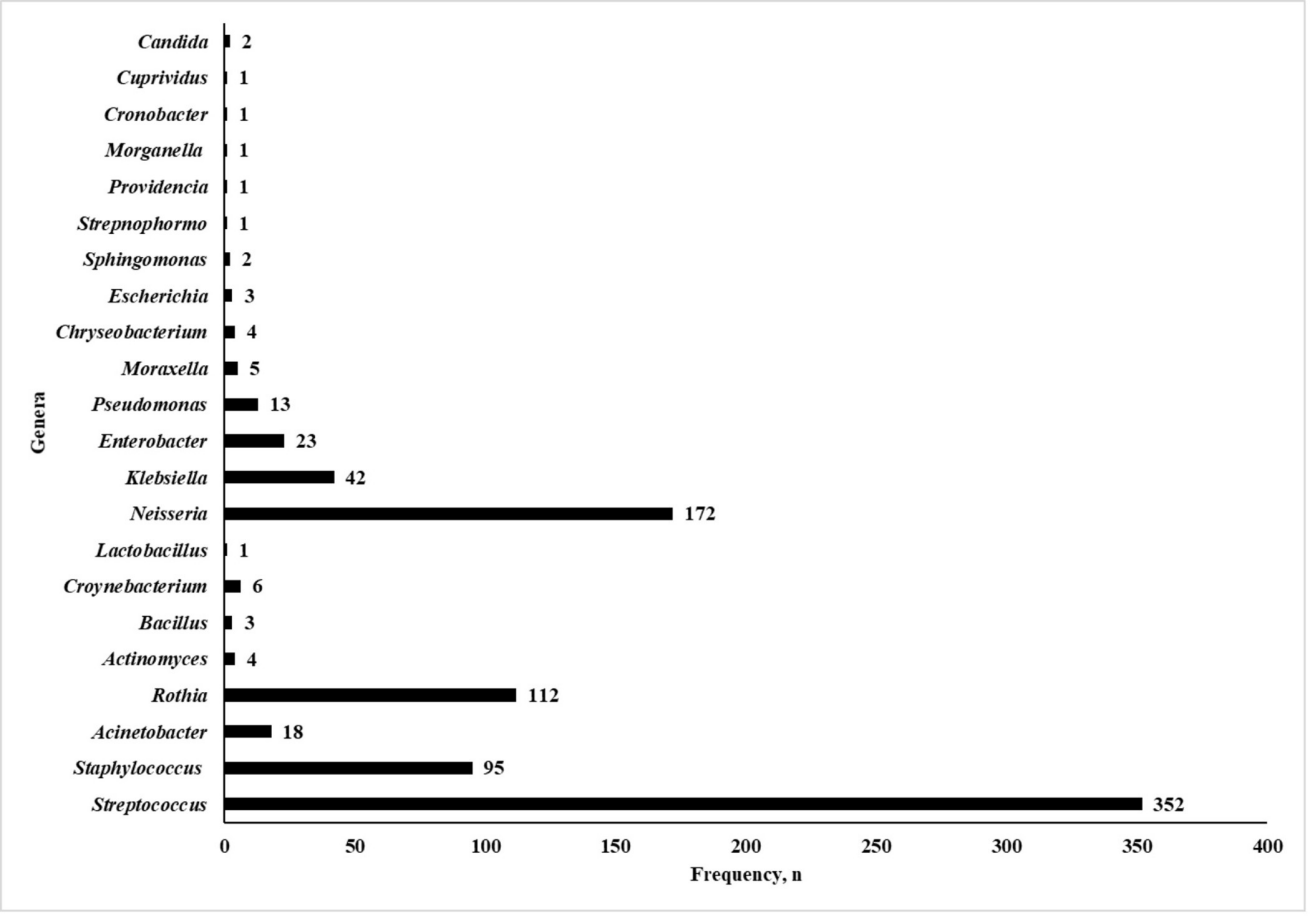
Frequency of genera with non-repetitive isolates found in AP patients’ throat.

A total of 22 genera were identified in the pharyngeal microbiome of 205 AP patients (Fig 1). From this total, *Streptococcus* constitutes the most prevalent bacteria genus (40.9%) in this study of 205 acute pharyngitis patients, followed by *Neisseria* (20%), *Rothia* (13%), *Staphylococcus* (11%) and *Klebsiella* (4.9%). The frequency of microorganisms identified from 205 AP patients is listed in Table 5.

**Table 5:** Frequency of microorganisms isolated from acute pharyngitis patient.

	Genera	Frequency, n (%)
**Gram-positive bacteria**	*Streptococcus*	352, (40.9)
	*Rothia*	112, (13)
	*Staphylococcus*	95, (11)
	*Actinomyces*	4, (0.5)
	*Bacillus*	3, (0.3)
	*Corynebacterium*	6, (0.7)
	*Lactobacillus*	1, (0.1)
**Gram-negative bacteria**	*Neisseria*	172, (20)
	*Klebsiella*	42, (4.9)
	*Enterobacter*	23, (2.7)
	*Acinetobacter*	18, (2.1)
	*Pseudomonas*	13, (1.5)
	*Moraxella*	5, (0.6)
	*Chryseobacterium*	4, (0.5)
	*Escherichia*	3, (0.3)
	*Sphingomonas*	2, (0.2)
	*Strepnophormo*.	1, (0.1)
	*Providencia*	1, (0.1)
	*Morganella*	1, (0.1)
	*Cronobacter*	1, (0.1)
	*Cuprividus*	1, (0.1)
**Yeast/Fungi**	*Candida*	2, (0.2)

The top 5 species in the acute pharyngitis microbiome are: *Streptococcus salivarius* (n = 128, 14.8%), *Neisseria flavescens* (n = 101, 11.7%), *Rothia mucilaginosa* (n = 90, 10.4%), *Staphylococcus aureus* (n = 78, 9.0%) and *Streptococcus pneumoniae* (n = 60, 7.0%). Interestingly, the bacterial etiological agent of AP warranting antibiotic prescription, Group A beta-hemolytic streptococci or *Streptococcus pyogenes* (GABHS) was found in only 5 AP patients in this study.

### Antibiotic-resistant bacteria clinical isolates found in AP patients

**Fig 2:**
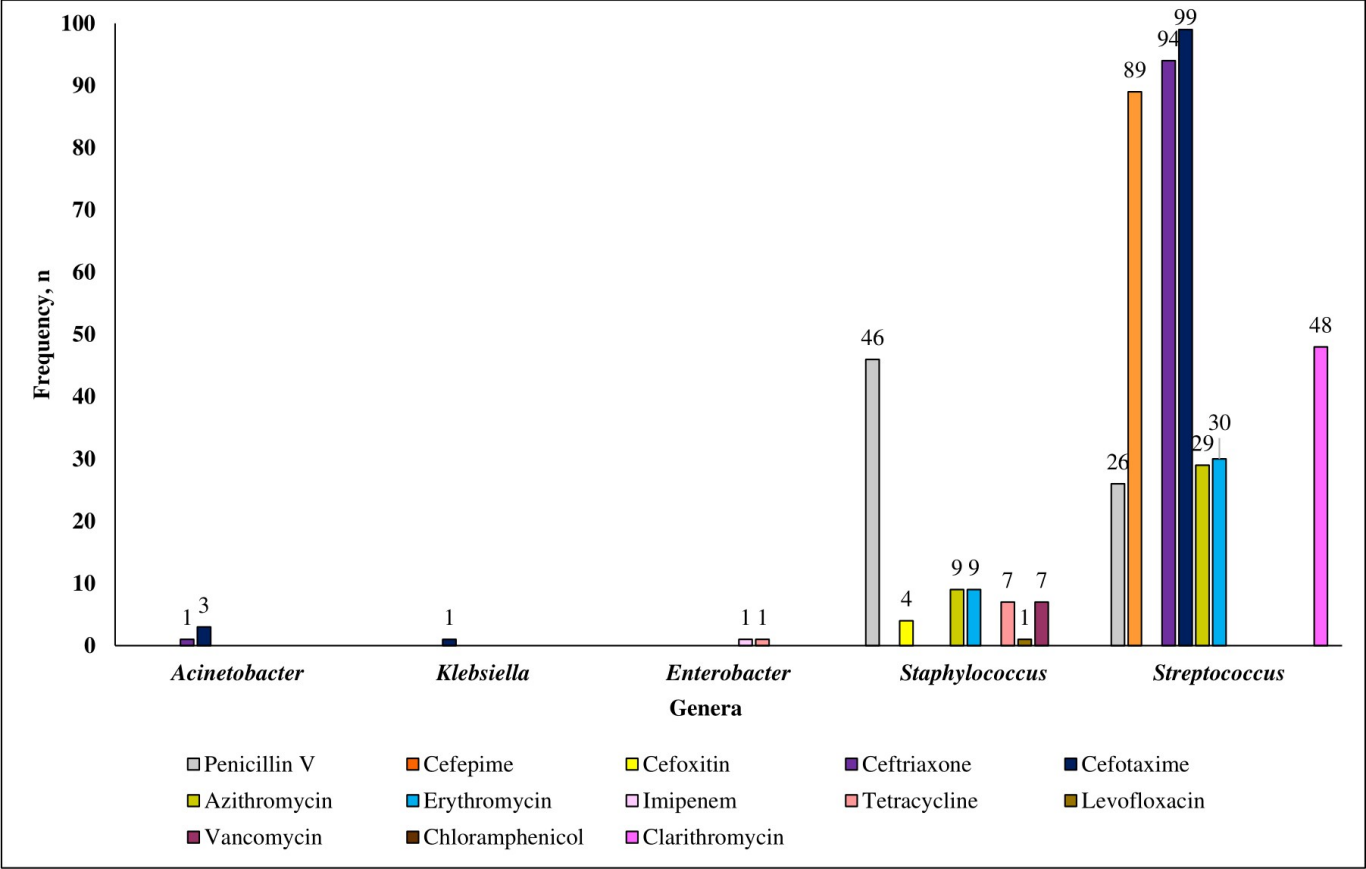
Antibiotic-resistant non-repetitive isolates found in AP patients.

Overall, Gram-positive bacteria were found to be resistant to more antibiotics tested than Gram-negative bacteria. The AST results showed that the *Streptococcus* and *Staphylococcus* genera were resistant to most of the antibiotics tested in this study (7 out of 19 types of antibiotics) (Fig 2). Of the antibiotics used in this study, resistance to cefotaxime was found in 10 different bacteria species, followed by resistance to ceftriaxone in 9 bacteria species, and resistance to azithromycin and erythromycin in 6 bacteria species.

Out of the 205 AP patients, 111 were found to carry at least one antibiotic-resistant bacteria species, where within this group, 76.6% (n = 85), 75.7% (n = 84) and 55.9% (n = 62) of them carried resistant bacteria towards cefotaxime, ceftriaxone and penicillin V antibiotics respectively. Alarmingly, we report findings of vancomycin-resistant *S*. *aureus* (VRSA) (n = 9), a first from the Malaysian community setting, where 5 out of these 9 VRSA isolates were also found to be methicillin-resistant *S*. *aureus* (MRSA) and one of these 5 is multidrug resistant (MDR).

### Antibiotic prescription patterns and appropriateness by GPs

**Fig 3:**
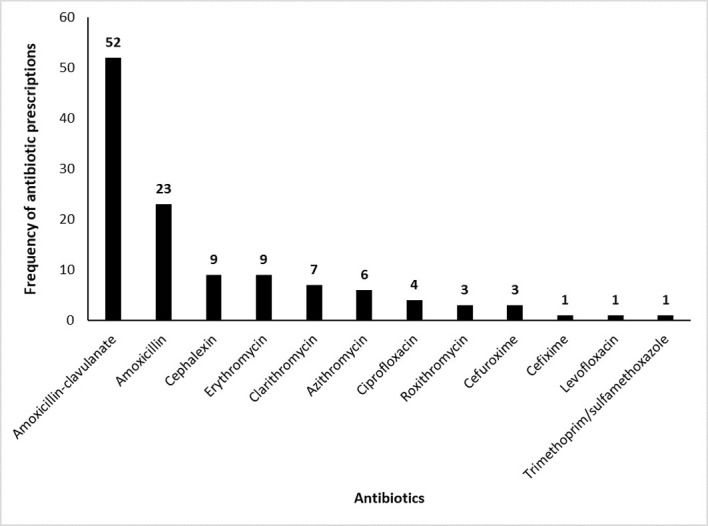
Types of antibiotics prescribed by GPs in this study.

More than half of the patients (n = 120/205) were prescribed with antibiotics with the frequency of antibiotic prescription by GPs is shown in Fig 3. Amoxicillin-clavulanate was the most commonly prescribed antibiotic received by AP patients in this study.

MALDIT-OF MS result has identified a total of 5 AP patients with non-repetitive GABHS-positive isolates and 4 out of 5 AP patients were prescribed with antibiotics. The overall appropriateness, including GABHS-positive patients who received antibiotics and GABHS-negative patients who did not receive antibiotics, was 42.9% (88/205) (Table 6), where 4 out of 5 GABHS-positive AP patients were prescribed antibiotics. The highest McIsaac score in this study is 4 and the antibiotic prescription appropriateness by applying post-diagnosis McIsaac scoring was able to rule out GABHS pharyngitis in this sample with a GABHS culture-positive sensitivity of 40% (n = 2/5) and specificity of 90% (180/200) (Table 7).

**Table 6:** Antibiotic prescription based on culture from throat.

Culture from throat swab	Were antibiotic prescribed?		Total
	Yes	No	
GABHS-positive	4	1	5 (2.4%)
GABHS-negative	116	84	200 (97.6%)
Total	120	85	205(100%)

**Table 7:** Antibiotic prescription appropriateness based on McIsaac score.

McIsaac score	Culture-positive	Culture-negative	Total	Risk of GABHS pharyngitis (%)*	Clinical recommendation*	Antibiotics prescribed?	
						Yes	No
0	0	79	79	1–2.5	No laboratorial investigation or antibiotic prescription	33	46
1	3	57	60	5–10	No laboratorial investigation or antibiotic prescription	29	31
2	0	44	44	11–17	Controversial	37	7
3	1	17	18	28–35	Antibiotic prescription	17	1
4	1	3	4	51–53	Antibiotic prescription	4	0
5	0	0	0	51–53	Antibiotic prescription	0	0
Total	5	200	205				

*Adapted from McIsaac *et al*. (2004) [25].

### Factors associated with antibiotic prescription by GPs

In this study, 58.5% (n = 120/205) of patients were prescribed antibiotics by the GPs. AP patients with sore throat lasting 49–72 hours had four times higher odds of being prescribed with antibiotics compared with those who had a sore throat duration of only 24 hours prior to consulting a doctor, while for those with more than 72 hours, the odds were six time higher. AP patients with symptoms or signs of fever, tonsillar swelling or exudate and runny nose were significantly associated with antibiotic prescription by GPs. The odds of AP patients given antibiotics for fever was 2.4 times higher than those without fever. The odds of AP patients being prescribed with antibiotics by GPs with the presence of tonsillar swelling or exudate was 5.8 times higher than those without tonsillar swelling or exudate. The odds of AP patients with runny nose being prescribed with antibiotics by GPs was 0.4 times odds of being prescribed antibiotics among those without runny nose (Table 8).

**Table 8:** Risk factors associated with antibiotic prescription using Generalized Estimating Equation Logit modeling.

		B coefficient	Std. Error	Sig.	Exp (B)	95% Confidence Interval	
						Lower	Upper
**Age**		-0.011	0.0189	0.567	1	0.953	1.027
**Gender**	**Female**				1		
	**Male**	0.655	0.3799	0.085	2	0.914	4.054
**Ethnic**	**Malay**				1		
	**Chinese**	0.652	0.5232	0.213	2	0.688	5.350
	**Others**	-0.364	0.6483	0.574	0.7	0.195	2.476
**Antibiotic use within last 12 months**	**Yes**	0.653	0.3948	0.098	2	0.886	4.165
	**No**				1		
**Duration of current sore throat**	**Within 24 hours**				1		
	**25–48 hours**	0.670	0.4249	0.115	2	0.085	4.493
	**49–72 hours**	1.411	0.5410	0.009*	4.1	1.420	11.84
	**> 72 hours**	1.815	0.7044	0.010*	6.1	1.544	24.416
**Symptoms**	**Fever >38°C**	0.870	0.3967	0.028*	2.4	1.097	5.193
	**Cough**	0.425	0.3965	0.283	1.5	0.704	3.328
	**Swollen anterior cervical nodes**	1.072	0.7606	0.159	2.9	0.658	12.969
	**Tonsillar swelling or exudate**	1.751	0.4553	0.000*	5.8	2.360	14.060
	**Oropharyngeal ulceration**	0.022	0.8688	0.980	1	0.186	5.610
	**Malaise**	0.340	0.3747	0.364	1.4	0.674	2.928
	**Runny nose**	-0.801	0.3892	0.040*	0.4	0.209	0.963
	**Hoarseness**	0.258	0.4537	0.569	1.3	0.532	3.151
	**Diarrhea**	-0.968	0.8549	0.257	0.4	0.071	2.029
	**GI symptoms**	-0.190	0.9075	0.834	0.8	0.140	4.897
	**Headache**	0.300	0.3756	0.424	1.4	0.647	2.818

*Note*. *p<0.05.

## Discussion

Our study found that in AP patients, *Streptococcus* (40.9%), followed by *Neisseria* (20.0%), *Rothia* (13.0%), *Staphylococcus* (11.0%), *Klebsiella* (4.9%), *Enterobacter* (2.7%), *Acinetobacter* (2.1%) and *Pseudomonas* (1.5%), are the top 7 most prevalent bacteria. According to analysis of the pharyngeal microbiome within the Human Microbiome Project, Gao *et al*. [35] reported the top 7 most common genera (in descending order) as *Prevotella*, *Capnocytophaga*, *Campylobacter*, *Veillonella*, *Streptococcus*, *Neisseria* and *Haemophilus*. Our study did not investigate anaerobic bacteria profiles and thus was not able to identify species such as those of the genera *Veillonella*. Nonetheless, there is a potential shift in the general landscape of microflora towards *Streptococcus* and *Neisseria* as the predominant genera in the AP pharyngeal microbiome. High rates of antibiotic-resistant bacteria were observed in urinary tract infections in primary care in the community in several Asian regions such as Indonesia, Hong Kong and Singapore [36–38], however, the overall availability of published surveillance data on antibiotic resistance in upper respiratory infections, specifically acute pharyngitis in the community is limited.

The finding of VRSA in the Malaysian community is disturbing as vancomycin has been recommended as first-line therapy for MRSA infections in Malaysia [39]. The spread of VRSA outside the clinical setting may render vancomycin resistance a major problem for the treatment of MRSA and VRSA infections. From the present study, it is not known if the presence of VRSA bacteria influences the pharyngeal microbiota diversity. However, what literature has shown is that *S*. *aureus* in throat carriage has been frequently reported [40–43]and community-associated MRSA is able to be transmitted from humans to animals and back into humans [44] while Richards *et al*. [45] revealed that the antibiotic-resistant genes in bacteria found in humans were able to be transmitted to animals. Therefore, we propose that the presence of VRSA in the community could increase the risk of transferring the pathogen or its resistant genes to pet or livestock animals in the food chain, as well as into the environment.

About 50% to 80% of acute pharyngitis symptoms are viral in origin and including a diversity of viral pathogens, predominantly rhinovirus, influenza, adenovirus, coronavirus, and parainfluenza [46]. In the present study, the predominant viruses are rhinovirus (hRV) and adenovirus (hAdV), the prevalence of infection was similar for both sexes, all ethnicity and age groups (Table 4). Thus, there does not seem to be any predilection for sex, ethnicity or age—both sexes, all ethnicity groups and all age groups are equally affected in this study. Both viruses are commonly found in the environment and can be transmitted via fecal-oral or direct person-to-person and consumption of contaminated water and food [47,48]. The possible reason for the predominance of hRV and hAdv might due to the high temperature and relative humidity in Malaysia that favors the transmission of these viruses as hAdv and hRV were more stable and survive best at high relative humidity [49,50]. To date, extensive studies of the viral etiology and epidemiology of respiratory tract infections have been conducted among pediatric populations rather than adult populations throughout the world [51–53]. A similar situation was seen in Malaysia whereby data regarding viral etiology of respiratory infections among adult patients in Malaysia is noticeably absent. A recent study has reported that rhinovirus or enterovirus was the common respiratory virus detected in adults with severe acute respiratory infections prior to COVID-19 pandemic in Malaysia [54]. Furthermore, according to Chong et al. [55], less than 5% of adult COVID positive patients in Malay were found be co-infected with rhinovirus/enterovirus. Nonetheless, it could be deduced that the respiratory viruses cause disease throughout the year in tropical country such as Malaysia.

The most common bacterial etiological agent of AP is GABHS, which causes 5% to 36% of cases of AP [46,56]. GABHS AP typically has an acute onset, lacks symptom of a viral URTI such as a cough, and is associated with fever, tonsillar exudates, and cervical adenopathy [46]. Pharyngitis caused by GABHS, or *Streptococcus* pyogenes, is concerning owing to its associated severe complications such as acute rheumatic fever and glomerulonephritis [56]. In the present study, only 2.4% (n = 5) of AP were GABHS culture-positive, which is lower than in literature, possibly due to the small sample size. It is also possible that Malaysia has an inherently low prevalence of GABHS AP. Malaysia has thus so far not have data on AP prevalence and aetiology in the adult population, and the large variation in the prevalence of GABHS in AP from one country to another, both in Asian and Western countries, has been acknowledged by the Clinical Practice Guidelines on Management of Sore Throat in Malaysia [11]. Moreover, GABHS culturing is not routinely performed in primary care, therefore, the GPs might not be well-trained for collecting throat swabs. As a result, these might affect the bacterial load in throat swabs which the presence of GABHS could be missed out during culturing. Presence of *Neisseria* species and *Streptococcus* species is suggested to play a role in offering protection from acquisition of GABHS infection. According to Crowe et al. [57], a few organisms in throat microflora were found to interfere with colonization of GABHS by exhibiting bactericidal activity, among these organisms were viridans streptococci and *Neisseria* spp. A few other studies proved similar [58,59]; interfering capability of viridans streptococci was especially apparent in Sanders [60]’s study where viridans *Streptococci* was the sole microorganism that antagonized growth of GABHS among four indigenous organisms studied (*Streptococcus* spp. viridans group, *Staphylococcus epidermis*, *Diphtheroids* and non-pathogenic *Neisseria*).

Antibiotic treatment is of no proven benefit for AP of bacterial origin other than GABHS [61]. Ideally, the gold standard of taking a throat swab for culture to document the presence of GABHS should be done before starting empirical treatment with antibiotics, but the practical constraints such as economic cost and lack of accessibility, especially in the private primacy care setting, hinders proper management of antibiotic prescription. Drugs of choice for adult AP patients include penicillin V and erythromycin as first-line antibiotics of choice, followed by macrolides such as clarithromycin, first generation cephalosporins and clindamycin [11], where resistance may develop during treatment with azithromycin and clarithromycin, which is why they are not first-line antibiotics for AP [46]. Analysis of antibiotics prescribed in our study also found that penicillin V, which is also the recommended antibiotic for sore throat management listed in the National Antimicrobial Guidelines (NAG) [39] was not prescribed for any patient in this study, suggesting a poor compliance of with the clinical practice guidelines. In fact, it is very interesting that the broad-spectrum antibiotics amoxicillin or amoxicillin-clavulanate are the most common antibiotics prescribed instead, suggesting that a large proportion of antibiotics prescription could be inappropriate. Similar findings were reported by Ahmad *et al*. [62] with amoxicillin and amoxicillin-clavulanate being the choice of antibiotics for URTI patients in the emergency department of a Malaysian public tertiary hospital while Teng *et al*. [14] reported that the main antibiotics chosen for URTIs in both public and private primary care were penicillins (47.7%) and macrolides (37.6%).

The main challenge of AP management is that fact that it is a very common reason for potentially inappropriate contact with antibiotics in the community and the potential for the community itself to be a self-propagating reservoir for antibiotic resistance throughout the ecosystem. Thus, the McIsaac scoring was developed to circumvent the need for GABHS testing in each presentation of AP to ascertain antibiotic prescription appropriateness. The Malaysian Clinical Practice Guidelines for Sore Throat Management [11] recommends the use of the McIsaac coring system, which is based on age as well as 4 clinical symptoms of tonsillar swelling or exudates, swollen anterior cervical nodes, fever greater than 38°C and a lack of cough [25]. The likelihood of a patient having GABHS infection increases with the total score, which will be used to assist medical personnel decisions in antibiotic prescription, where a score of 3 to 5 warrants antibiotic prescription.

Analysis of our results found that patients who had a fever of greater than 38°C or tonsillar swelling or exudates have higher odds of being prescribed antibiotics, even if they did not fulfil McIsaac scores of 3, 4 or 5. Linder and Singer [63] have also found that fever and pharyngeal exudate were the significant predictors for antibiotic prescription for URTI in the USA. We also found that patients with absence of runny nose in our study had significantly lower odds of being prescribed with antibiotics. The probable explanation for this is that the presence of runny nose is more suggestive of viral upper respiratory tract infection and the presence erythema, swelling and fever are more likely of bacterial pharyngitis infection [64,65], which agrees with McIsaac’s recommendations, but nevertheless still sparks the question of why AP patients who do not present with symptoms of GABHS are also being prescribed antibiotics in the private primary care setting. In the present study, the overall antibiotic prescription proportion was 58.5%, although only 2.4% of our AP patients were GABHS culture-positive, and only 10.7% were scored as 3 or 4 on the McIsaac score post-diagnosis, with none scored as 5. Thus, the antibiotic prescription proportion is not attributable to the presentation of GABHS AP symptoms. The antibiotic prescription pattern seen in the present study is even higher than the 46.7% as reported by Teng *et al*. [66] for URTIs in private primary care, suggesting the failure of compliance to the Malaysian Clinical Practice Guidelines on Sore Throat Management [11] despite its introduction more than a decade ago.

Noticeably, the high antibiotic prescribing patterns for URTIs seen in primary care settings are not only common in Malaysia, but also in other Asian countries as well, including China (83.7%), Japan (60.0%) and India (57.0%) [67–69]. Similarly, high antibiotic prescribing patterns of 67.2% and 57.7% were observed in previous studies in the Malaysian private sector, reported by Ab Rahman *et al*. [15] and Teng *et al*. [64]. Prescriber behavior is an important reason for antibiotic utilization. In our study, the private primary care practitioners (GPs) were not informed to follow Clinical Practice Guidelines but were left to freely make their own diagnosis and prescriptions. Thus, the GPs could be unaware of the McIsaac scoring system to assess the likelihood of GABHS AP, even though management recommendations of sore throat are within the Malaysian Clinical Practice Guidelines on Sore Throat Management [11] and National Antimicrobial Guidelines [39], or other factors. As Hassali *et al*. [70] reported previously, most private primary care practitioners (or general practitioners, GPs) in Selangor, Malaysia stated they would “sometimes” prescribe antibiotics to sore throat patients even though most of the cases were likely viral pharyngitis. Ab Rahman *et al*. [15] suggests that this could be due GPs being responsive to the patients’ expectation for antibiotics upon consultation.

The McIsaac scoring system is an effective no-cost method towards managing AP in Malaysia in terms of reducing inappropriate antibiotic prescription. Thillaivanam *et al*. [71] reported that, in a pediatric ward in a Malaysian public hospital, with the enforcement of the McIsaac clinical decision rule a significantly reduction (by 26.5%) of antibiotic prescription was observed, while a successful improvement (by 22.9%) of prescribers’ compliance to the Malaysian Clinical Practice Guideline for Sore Throat was also seen In the interest of a simple, cost-effective and rapid method, the McIsaac scoring system could be used by GPs as the proportion of inappropriate prescription of antibiotics for this study could be reduced from 82.5% (99/120) to only 17.5% (21/120) with the application of retrospective McIsaac scoring based on the symptoms observed by the GPs. Thus, GABHS pharyngitis can be successfully ruled out in this study by applying the McIsaac decision rule of an antibiotic prescribing score ≥3, which gives a sensitivity of 40% and specificity of 90%. Assessment of the challenges GPs face in applying the McIsaac scoring system may improve antibiotic prescription appropriateness in the private primary care sector. In mitigation of continuous inappropriate antibiotic prescription in the community setting, we suggest conducting more awareness programs on antibiotic consumption and prescription targeting not only the general public but also health care providers through continuous medical education (CME).

The present study has a number of important features, whereby only AP patients were included while other studies usually include patients with URTIs such as rhinitis, cough, otitis media and common cold apart from pharyngitis. Our study also focused on the antibiotic resistance profiles of the throat microflora rather than only on GABHS-positivity and thus may provide useful information on the prevalence of antibiotic-resistant strains that could potentially cause invasive diseases. In addition, we have presented this study on AP patients in a Malaysian private primary care setting, which could serve as reference data in monitoring the antibiotic resistance patterns among the Malaysian community. That being said, the temporal relationships in a cross-sectional study such as this are ambiguous and makes causal inference difficult. In addition, the results may not be generalizable to public hospitals and clinics. Another limitation of the study is the small sample size that could cause large standard errors in the B coefficients, and hence, large 95% confidence intervals for the odds ratios. Also, the convenience sampling technique used in this study might be a limiting factor that could lead to biased estimates of antibiotic resistance and prescription practices among the GPs. Also, there was some detection bias in this study whereby only culturable microorganisms were identified via MADTI-TOF mass spectrometry. The virus detection used in this study was limited to detection of viral DNA and RNA but without cause and effect; on the other hand, culture method was used for bacteria resistance profiling. Other studies for detection of pathogens in URTIs were done using microbiome analysis using 16S or Next Generation Sequencing (NGS) and metagenomic analysis [72–74]. Therefore, we suggest that a combination of non-culturable method such as microbiome study with a culturable method for cause-and-effect analysis could be carried out in future study.

## Conclusions

In conclusion, the majority of acute pharyngitis patients in the present study are likely of viral aetiology, with rhinovirus and adenovirus as the predominant viruses. Community-based *S*. *aureus* resistant to vancomycin (VRSA) and also to multiple antibiotics (MDR-VRSA) were identified in this study. The most common antibiotic prescribed was amoxicillin or amoxicillin-clavulanate, not the recommended first-line penicillin V. A high proportion of antibiotic prescription was seen in this study with the majority being inappropriate for acute pharyngitis showing symptoms of viral pharyngitis. Inappropriate antibiotic prescription was reduced when McIsaac scoring was applied, suggesting that appropriate antibiotic prescription for acute pharyngitis among private primary care practitioners can be improved with the simple use of the McIsaac clinical decision rule during diagnosis. Future studies could involve using the cohort study design to minimize bias and maximize accuracy in the estimates of antibiotic resistance and antibiotic prescription. The behavioral factors of GPs and patients could be included in future studies to improve the predictive model in determining the possible factors that may contribute to inappropriate antibiotic prescription practices.

## Supporting information

S1 Table Antibiotics used in this study. (DOCX)

S2 Table The McIsaac scoring system to use for children and adults with a sore throat to estimate probability of GABHS infection. (DOCX)

## Abbreviations

AP: acute pharyngitis
GABHS: Group A beta-haemolytic *streptococcus*
GPs: general practitioners
