# Supplementary material for: Respiratory microorganisms in acute pharyngitis patients: Identification, antibiotic prescription patterns and appropriateness, and antibiotic resistance in private primary care, central Malaysia
Source: PLoS One. 2022 Nov 17;17(11):e0277802. doi: 10.1371/journal.pone.0277802 (PMC9671416; doi:10.1371/journal.pone.0277802)
Supplement: S1 Table — (DOCX) [file pone.0277802.s001.docx]

| Antibiotics | Concentration | Catalog/Batch number; Brand |
| --- | --- | --- |
| Azithromycin dihydrate | 15 μg | SMP/14170/AZI/23; Kotra Pharma, Malaysia |
| Phenoxymethylpenicillin potassium | 10 U | 20061501072; Kotra Pharma, Malaysia |
| Erythromycin ethyl succinate | 15 μg | SMS/15009/ERY/01; Kotra Pharma, Malaysia |
| Cefepime | 30 μg | CT077B; Oxoid, UK |
| Cefotaxime | 30 μg | CT0166B; Oxoid, UK |
| Cefoxitin | 30 μg | CT0119B; Oxoid, UK |
| Ceftriaxone | 30 μg | CT0417B; Oxoid, UK |
| Clarithromycin | 15 μg | CT0693B; Oxoid, UK |
| Imipenem | 10 μg | CT0455B; Oxoid, UK |
| Meropenem | 10 μg | CT0774B; Oxoid, UK |
| Ciprofloxacin | 5 μg | CT0425B; Oxoid, UK |
| Levofloxacin | 5 μg | CT1587B; Oxoid, UK |
| Chloramphenicol | 30 μg | CT0113B; Oxoid, UK |
| Tetracycline | 30 μg | CT0119B; Oxoid, UK |
| Cefoxitin | - | T1023; Targetmol, US |
| Cefotaxime | - | T0911; Targetmol, US |
| Ceftriaxone | - | T1223; Targetmol, US |
| Imipenem | - | T1505; Targetmol, US |
| Levofloxacin | - | T6567; Targetmol, US |
| Tetracycline | - | T0912L; Targetmol, US |
| Vancomycin hydrochloride | - | VWR, US |

S1 Table. Antibiotics used in this study
