# Supplementary material for: Respiratory microorganisms in acute pharyngitis patients: Identification, antibiotic prescription patterns and appropriateness, and antibiotic resistance in private primary care, central Malaysia
Source: PLoS One. 2022 Nov 17;17(11):e0277802. doi: 10.1371/journal.pone.0277802 (PMC9671416; doi:10.1371/journal.pone.0277802)
Supplement: S2 Table — (DOCX) [file pone.0277802.s002.docx]

| **Criteria** | **Points** |
| --- | --- |
| Fever (temperature > 38 ⁰C) | + 1 |
| Absence of cough | + 1 |
| Swollen and tender anterior cervical nodes | + 1 |
| Tonsillar swelling or exudates | + 1 |
| Age (years) | |
| 3 – 14 | + 1 |
| 15 – 44 | 0 |
| > 45 | - 1 |

S2 Table. The McIsaac scoring system to use for children and adults with a sore throat to estimate probability of GABHS infection. [Adapted from McIsaac et al. (2004)]

Risk for GABHS pharyngitis. [Adapted from McIsaac *et al*. (2004)]

| **Score** | **Risk (%)** | **Clinical recommendation** |
| --- | --- | --- |
| < 0 | 1 – 2.5 | No laboratorial investigation or antibiotic prescription |
| 1 | 5 – 10 | No laboratorial investigation or antibiotic prescription |
| 2 | 11 – 17 | Controversial |
| 3 | 28 – 35 | Controversial |
| > 4 | 51 – 53 | Antibiotic prescription |
